# Use of Wild Type or Recombinant Lactic Acid Bacteria as an Alternative Treatment for Gastrointestinal Inflammatory Diseases: A Focus on Inflammatory Bowel Diseases and Mucositis

**DOI:** 10.3389/fmicb.2017.00800

**Published:** 2017-05-09

**Authors:** Rodrigo D. De Oliveira Carvalho, Fillipe L. R. do Carmo, Alberto de Oliveira Junior, Philippe Langella, Jean-Marc Chatel, Luis G. Bermúdez-Humarán, Vasco Azevedo, Marcela S. de Azevedo

**Affiliations:** ^1^Federal University of Minas Gerais – Instituto de Ciências BiológicasBelo Horizonte, Brazil; ^2^Micalis Institute, Institut National de la Recherche Agronomique, AgroParisTech, Université Paris-SaclayJouy-en-Josas, France

**Keywords:** inflammatory bowel diseases, mucositis, lactic acid bacteria, *Lactococcus lactis*, genetic engineering

## Abstract

The human gastrointestinal tract (GIT) is highly colonized by bacterial communities, which live in a symbiotic relationship with the host in normal conditions. It has been shown that a dysfunctional interaction between the intestinal microbiota and the host immune system, known as dysbiosis, is a very important factor responsible for the development of different inflammatory conditions of the GIT, such as the idiopathic inflammatory bowel diseases (IBD), a complex and multifactorial disorder of the GIT. Dysbiosis has also been implicated in the pathogenesis of other GIT inflammatory diseases such as mucositis usually caused as an adverse effect of chemotherapy. As both diseases have become a great clinical problem, many research groups have been focusing on developing new strategies for the treatment of IBD and mucositis. In this review, we show that lactic acid bacteria (LAB) have been capable in preventing and treating both disorders in animal models, suggesting they may be ready for clinical trials. In addition, we present the most current studies on the use of wild type or genetically engineered LAB strains designed to express anti-inflammatory proteins as a promising strategy in the treatment of IBD and mucositis.

## Introduction

The gastrointestinal tract (GIT) is colonized by a complex community of microorganisms, known as the intestinal microbiota, consisting mainly of bacteria that are classified as indigenous or transient. Symbiotic bacteria, such as short chain fatty acid (SCFA)-producing species from the Lactobacillales order and *Faecalibacterium prausnitzii*, contribute to host metabolism and immune system function while occupying a protected environment rich in nutrients (Hooper and Macpherson, 2010; de Vos and de Vos, 2012; Chang and Lin, 2016). Pathobionts of the GIT, consisting mainly of Proteobacteria such as *Escherichia coli* and *Clostridium difficile*, present a potential risk to the GIT by disrupting the integrity of tissues if, for instance, they are allowed to grow in number (Lebeer et al., 2010; Vangay et al., 2015).

Therefore, the host contains several biological structures that are essential for controlling bacterial overgrowth and invasion. In this context, the mucous layer protecting the intestinal epithelial cells (IECs) plays an important role by restricting the contact of harmful bacteria with host cells (Johansson et al., 2013; Peterson and Artis, 2014). In addition, specialized IEC, such as Paneth cells, secrete several antimicrobial peptides to eliminate microbes that eventually penetrate into the mucus (Salzman et al., 2007; Carlsson et al., 2013). When pathobionts translocate into the intestinal epithelium, the host immune response is activated to eliminate them by producing pro-inflammatory mediators. However, the overproduction of these compounds represents a risk, as they can inflame the tissue, causing intestinal barrier disruption and mucosal dysfunctions in the host (Hidalgo-Cantabrana et al., 2014; Kashyap et al., 2014). Therefore, to maintain intestinal homeostasis, specialized immunological structures, known as the gut-associated lymphoid tissue (GALT), must be able to specifically recognize and eliminate the pathogenic species while tolerating the commensals (Izcue et al., 2009; Carlsson et al., 2013).

Under normal conditions, GALT generates tolerance to commensals mainly through the action of regulatory T (Treg) cells. When the dynamic balance between Treg and activated effector T cells is broken, homeostasis is compromised and may lead to the development of mucosal inflammation in the gut (Strober et al., 2007). In addition to microbiota composition impairment, known as dysbiosis, other factors can influence the proper functioning of the GIT immune system, including individual genetic susceptibility, diet, use of drugs and environmental stress (Ananthakrishnan, 2015). The intersection of these factors may generate an exaggerated pro-inflammatory reaction against the microbiota that causes inflammatory bowel diseases (IBDs), a group of idiopathic and chronic inflammatory conditions of the GIT, which primarily includes ulcerative colitis (CD) and Crohn’s disease (UC) (Vangay et al., 2015; Velasquez-Manoff, 2015). In addition, other factors, such as the use of some medications, can also contribute to the breakdown of this immunological tolerance against commensals. It has been reported that chemotherapeutic agents, such as 5-fluoracil, that are widely used in the treatment of advanced solid tumors, may also lead to the development of another inflammatory condition of the GIT known as mucositis, a disease characterized by painful inflammation and ulceration of the mucosal membranes (Soares et al., 2013; Pedroso et al., 2015).

CD and UC are associated with severe intestinal inflammation, and patients have reported gastrointestinal (GI) symptoms such as abdominal pain, diarrhea, rectal bleeding, and weight loss (Lennard-Jones, 1989; Stepaniuk et al., 2015). IBD represent a global health issue, as its incidence has increased in several countries, while safe and efficient therapies are still in development (Molodecky et al., 2012; Ananthakrishnan, 2015). Mucositis induced by 5-FU is of great clinical significance as well, as it might result in cancer therapy being adjusted, affecting a patient’s chances of survival (de Vasconcelos Generoso et al., 2015; Antunes et al., 2016). Thus, the scientific community has sought novel therapeutic alternatives to fight both IBD and mucositis. As dysbiosis plays a key role in the pathogenesis of both diseases, the modulation of the patient microbiota via the administration of probiotic bacteria has been proposed.

## Use of Probiotic Lactic Acid Bacteria in the Treatment of Gastrointestinal Inflammation

Over a century ago, Elie Metchnikoff was the first to propose the rationale for using host-friendly bacteria found in yogurt to manipulate the intestinal microbiome. He also predicted the existence of bacterial translocation, from the intestinal lumen to inner layers of the mucosa and also to systemic organs, and described theories associating the microbiota with intestinal inflammation and other diseases (Mackowiak, 2013). Currently, several research groups have confirmed his hypothesis, demonstrating that the administration of certain bacterial species in several animal models actually provides health benefits to alleviate inflammation, including the containment of inflammatory mediators, stimulation of the immune system and microbiota restoration by competitive exclusion of potentially pathogenic species (Ljungh and Wadström, 2006; Luerce et al., 2014; Quinto et al., 2014; Santos Rocha et al., 2014; Thomas, 2016). These microorganisms are considered to be probiotics, a term defined by the World Health Organization (WHO) as “live microorganisms administered in adequate amounts that confer a beneficial health effect on the host” (FAO/WHO, 2002).

Probiotics are live bacteria and yeasts; however, the majority of strains are gram-positive bacteria belonging to the *Lactobacillus*, *Bifidobacterium*, *Streptococcus*, and *Lactococcus* genera. These genera are included in a diverse group of microorganisms entitled lactic acid bacteria (LAB), as they are able to convert sugars into lactic acid (Holzapfel et al., 1998; Carr et al., 2002). With regards to Gram-negative bacteria, some strains of *E. coli* are also considered to promote health, for instance, *E. coli* Nissle 1917 (EcN1917) was originally isolated from the feces of a soldier during the First World War who did not develop infectious diarrhea during an outbreak of contagious Shigella (Westendorf et al., 2005; Henker et al., 2007).

Although Metchnikoff introduced the concept of probiotics in 1907, some of these microorganisms have been used for centuries to prepare yogurt, sourdough bread, sauerkraut, cucumber pickles and olives, as they are able to produce lactic acid, as previously mentioned (Mackowiak, 2013; Vikhanski, 2016). In the latter half of the 20th century, probiotics have gained visibility as there has been increasing interest in applying them to other areas, such as the pharmaceutical industry. Thus, the selection of new probiotic strains, the development of new food products based on probiotics and freeze-dried probiotic pharmaceutical formulations has increased in importance. There are many studies being conducted that focus on the development of probiotic-based pharmaceutical formulations that can be administered to either the gastrointestinal, nasal, or vaginal mucosa, as well as to the skin of patients (Guglielmetti et al., 2010; Iannitti and Palmieri, 2010; Vicariotto et al., 2012).

### The Lactic Acid Bacteria Group

The LAB group includes a heterogeneous group of ubiquitous microorganisms that obtain energy through the conversion of sugars into lactic acid. Morphologically, LAB bacteria can resemble cocci, rods, or bacilli. They are gram-positive microorganisms with a low genomic GC content (54%) and are facultative anaerobes that are non-spore-forming, immotile and do not produce catalase (Stiles and Holzapfel, 1997; Carr et al., 2002). Species of this group can be naturally found in different environments that are rich in nutrients, such as decomposing vegetables and fruits, and even in the oral, urogenital and intestinal tracts of mammals and other animals. They can also be found in several kinds of dairy foods, as some strains are used to produce them (Holzapfel et al., 1998; Liu et al., 2014). LAB species found in the human GIT can be autochthonous as indigenous GI microflora, especially those belonging to the *Lactobacillus* and *Streptococcus* genera, or allochthonous as transients of the GIT, such as *Lactococcus* sp. and some strains of *Lactobacillus* used to produce yogurts. Some species, especially those belonging to the *Streptococcus* genera are pathogenic; however, the vast majority of LAB strains have a positive impact on human health and are generally regarded as safe (GRAS) by the United States Department of Agriculture (USDA) (Felis and Dellaglio, 2007).

After the pioneering work of Elie Metchnikoff, who first suggested that the ingestion of dairy foods produced by LAB fermentation could prevent intestinal infections and promote both health and human longevity, the scientific community is continuously exploring in more detail the positive effects promoted by these bacteria (Johnson and Klaenhammer, 2014; Vikhanski, 2016). Among all LAB species described that exert probiotic effects, *Lactobacillus* spp., *Streptococcus* spp., and *Lactococcus* spp. stand out for use in therapeutic applications for both the treatment and prevention of various intestinal disorders (Majamaa and Isolauri, 1997; Ouwehand et al., 2002; Prescott and Björkstén, 2007; Ohland and MacNaughton, 2010; Luerce et al., 2014; Santos Rocha et al., 2014). This topic has been widely studied, and certain immunological aspects of LAB anti-inflammatory properties have been described.

### Effects of Probiotic Lactic Acid Bacteria in Animal Models of Gastrointestinal Inflammation

**FIGURE 1:**
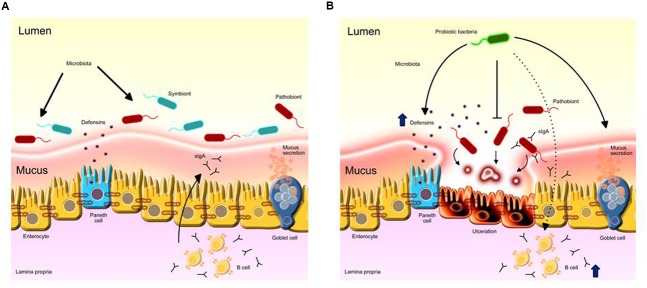
**Probiotic LAB anti-inflammatory mechanisms on the intestinal mucosa. (A)** Intestinal homeostasis provided by the healthy microbiota role in stimulating ephitelial barrier components such as mucus, Paneth cells activity and eliciting protective immune responses such as IgA. **(B)** The overgrowth of pro-inflammatory mucin-degrading pathobionts induces inflammation in the mucosa. Administration of probiotics prevents inflammatory responses by inhibiting the growth of pathogens directly; increases mucus secretion by goblet cells and the secretion of defensins by Paneth cells; fortificates tight junction stability; stimulates mucosal immunity by inducing IgA production by B cells to the intestinal lumen, limiting harmful microbe adherence and colonization.

Lactic acid bacteria probiotic strains can alleviate intestinal inflammation through several mechanisms (**Figure 1**). Accumulating evidence has revealed that probiotic LAB are able to protect the host against potentially pathogenic species that inhabit the GIT of animals, including humans. It seems that lactobacilli strains, such as *L. acidophilus* LA1, can prevent the colonization of the intestine by pathogenic bacteria, such as *Staphylococcus aureus, Salmonella typhimurium*, and *Pseudomonas aeruginosa*, by competitive exclusion (Bernet-Camard et al., 1997; Adolfsson et al., 2004). Apparently, these LAB compete for nutrients and adhesion sites in the intestinal epithelium with these potentially pathogenic bacteria that transit in the GIT and are consequently eliminated. The secretion of lactic acid and bacteriocins (natural antibiotics) by probiotic species has also been implicated in the mechanism of the elimination of pathogens (Ogawa et al., 2001; Moal et al., 2007).

Another manner by which LAB strains may protect the host from pathogen invasion is by boosting the intestinal epithelial barrier. Some LAB microbe-associated molecular pattern (MAMPs) are capable of interacting with epithelial pattern recognition receptors, mainly the Toll-like receptor-2 (TLR2), TLR6 and nod-like receptors (Ren et al., 2016). This activation induces several protective mechanisms that restore tissue damage, such as modulation of the stability of tight junctions (Lebeer et al., 2010; Ohland and MacNaughton, 2010; Villena and Kitazawa, 2014; Bajaj et al., 2015). Species such as *B. infantis*, *L. plantarum*, and *L. casei* have been shown to increase the expression of proteins involved in tight junction barrier function, such as occludins and zonula occludens-1 (ZO-1) (Ewaschuk et al., 2008; Anderson et al., 2010; Eun et al., 2011).

Some *Lactobacillus* strains are capable of increasing the production of other proteins involved in the maintenance of epithelial barrier homeostasis, such as mucin-2 (MUC2), the most abundant glycoprotein in mucus. *In vitro* studies showed that increased MUC2 expression in intestinal epithelial Caco-2 cells blocked the adhesion of pathogenic *E. coli* (Mattar et al., 2002; Mack et al., 2003). Furthermore, an *in vivo* study demonstrated that mice treated with a VSL#3 probiotic-mixture consisting of *S. thermophilus*, four strains of lactobacilli (*L. delbrueckii*, *L. casei*, *L. acidophilus*, and *L. plantarum*) and three species of *Bifidobacterium* (*B. longum*, *B. infantis*, and *B. breve*) for 7 days exhibited an approximate 60-fold increase in the production of MUC2 in treated animals (Gaudier et al., 2005).

Other studies have suggested that some LAB strains are able to induce the secretion of defensins by enterocytes, which are related to the biological control of potentially pathogenic species in the lumen. Administration of certain species of lactobacilli or the VSL#3 probiotic-mixture in mice resulted in an increase in the production of β-defensin-2, which has microbicidal activity against important opportunistic pathogens, such as *P. aeruginosa*, *E. coli*, and *Candida albicans* (Harder et al., 2004; Schlee et al., 2008).

The stimulation of the host immune system and the suppression of pro-inflammatory responses are well-established probiotic effects. One of the major mechanisms of these processes is the stimulation of immunological tolerance to GIT microbiota through an increase in IL-10 secretion and a significant reduction in IFNγ and IL-12 expression. This probiotic effect is caused due to the interaction of “good” bacteria with intestinal dendritic cells that drives the development of T regulatory cells and IgA-producing B cells (Fedorak et al., 2000; Ng et al., 2009). Administration of *B. lactis*, *B. bifidum*, and *B. infantis* in mice previously infected with rotavirus or enterohemorrhagic *E. coli* has been shown to increase the titers of specific IgA against the rotavirus (Shu and Gill, 2001; Qiao et al., 2002). For instance, Santos Rocha et al. (2014) showed that the probiotic effect of *L. delbrueckii* strain CNRZ327 was related to an expansion of Treg cells and an increase of total IgA in Dextran sulfate sodium (DSS)-induced colitis in mice. This effect was shown to be enough to prevent inflammation in mice (Santos Rocha et al., 2014). Recently, it was reported that a *Lactococcus lactis* ssp. *lactis* NCDO2118 strain prevented DSS-induced colitis in mice and the protective effect was related to increased IL-10 levels in the colon and the induction of Treg cells in the mesenteric lymph nodes (Luerce et al., 2014). In another study using a similar colitis model, *L. lactis* FC ssp. *cremoris* demonstrated a protective role in treating inflammation in mice, by preventing the NF-kB activation and in decreasing IL-8 expression in epithelial cells (Nishitani et al., 2009).

Lactic acid bacteria have also been studied and has generated promising results, both *in vitro* and *in vivo*, in other models of intestinal inflammation, such as preclinical mucositis models (Tooley et al., 2006; Bowen et al., 2007; Smith et al., 2008; Southcott et al., 2008; Whitford et al., 2009; Tooley et al., 2011; Prisciandaro et al., 2012). *In vitro*, it was observed that IECs previously treated with 5-FU presented reduced levels of cytotoxicity and apoptosis through the inhibition of caspase-3 and caspase-7 when co-cultured with *L. rhamnosus* (Prisciandaro et al., 2012). *In vivo*, *L. fermentum* BR11 administered to mice injected with 5-FU exhibited reduced levels of intestinal inflammation and myeloperoxidase enzyme activity, a marker of eosinophilic inflammation (Smith et al., 2008). In another study, VSL#3 was used in the treatment of mucositis that was induced in rats through the injection of a chemotherapy drug known as irinotecan. The administration of probiotics has been shown to prevent weight loss and reduce diarrhea in these rats. These findings were associated with significant improvement in the integrity of crypts in the jejunum and a reduction in apoptosis levels in both the small and large intestines of irinotecan-treated rats (Bowen et al., 2007). Whitford et al. (2009) compared the efficiency of live *S. thermophilus* TH-4 strain (TH-4), dead TH-4 and TH-4 culture supernatants in rats treated with 5-FU. They showed that live TH-4 significantly reduced disease severity scores as well as crypt fission indices, which is an indicator of longitudinal intestinal growth and stem cell proliferation, suggesting that this strain may be useful for treating diseases characterized by increased crypt fission, such as colorectal carcinoma. However, Tooley et al. (2011) ascertained the effects of live TH-4 on small intestinal damage generated by the injection of methotrexate (MTX), a chemotherapy drug that induces mucositis and tumor progression in tumor-bearing rats. This study verified that although TH-4 did not protect animals from chemotherapy-induced mucositis, the progression of mammary adenocarcinoma was unaffected (Tooley et al., 2011).

The efficacy of cow’s milk yogurt containing *L. johnsonii* and sheep’s milk yogurt containing *L. bulgaricus* and *S. thermophilus* was assessed in an MTX-induced model of mucositis in rats. It was shown that both types of yogurt reduced intestinal permeability, revealing them to be useful in restoring intestinal barrier function (Southcott et al., 2008).

## The Use of Recombinant Lactic Acid Bacteria for the Treatment of Git Inflammatory Diseases

As probiotics have been shown to be capable of acting on many diverse biological processes within the host, they have been experimented with as an alternative therapy against GIT inflammatory disorders. To enhance probiotic properties, research is focusing on the development of genetically modified bacterial strains expressing heterologous proteins of medical interest, such as anti-inflammatory molecules. Recently, the use of recombinant LAB strains with natural probiotic activities have shown promising results in pre-clinical studies as an alternative therapy to treat cancer, obesity, and especially GI tract inflammation (Bermúdez-Humarán et al., 2007; Cortes-Perez et al., 2007; Bahey-El-Din et al., 2010; Bermúdez-Humarán et al., 2013; Wang et al., 2016).

Since 1960, molecular biologists have developed several sophisticated techniques to identify, isolate, and manipulate the genetic components of the bacterial cell. This knowledge enabled the construction of different LAB recombinant strains with increased anti-inflammatory properties. Well-reported examples include the construction of *L. casei*, *L. plantarum*, *S. thermophilus*, and *L. lactis* strains capable of expressing anti-inflammatory molecules, thus increasing the benefitial effects of the above-mentioned strains (**Table 1**) (Han et al., 2006; LeBlanc et al., 2011; Del Carmen et al., 2014). Thus, several studies have focused on the use of recombinant anti-inflammatory LAB as an interesting alternative treatment for GIT inflammatory diseases (de Moreno de LeBlanc et al., 2015; Wang et al., 2016).

**Table 1:** Heterolgous proteins with anti-inflammatory properties produced in different strains of lactic acid bacteria.

Organism	Heterologous protein	Expression system	Inflammatory condition	Anti-inflammatory effects	Reference
*L. casei* BL23	Superoxide dismutase A from *L. lactis* MG1363	SodA native promoter from *L. lactis* MG1363	Mouse model of DSS-induced colitis	Protection against ROS	Watterlot et al., 2010
*L. fermentum* I5007	Superoxide dismutase from *B. subtilis*	Constitutive promoter from *L. casei* ATCC334	Mouse model of TNBS-induced colitis	Inhibition of NF-κB pathway	Hou et al., 2014
*S. thermophilus* CRL807	Superoxide dismutase A from *L. lactis* MG1363	SodA native promoter from *L. lactis* MG1363	Mouse model of TNBS-induced colitis	Reduction of intestinal permeability and histological damage	Del Carmen et al., 2014
*L. lactis* NCDO2118	Human 15-lipoxygenase-1	XIES	Mouse model of DSS-induced colitis	Decreased IFN-γ and IL-4. Increased IL-10	Carvalho et al., 2016
*L. lactis* NZ3900	Mouse cathelicidin	NICE	Mouse model of DSS-induced colitis	Reduced tissue damage and MPO activity	Wong et al., 2012
*L. lactis* NZ9000	Human elafin	NICE	Mouse model of DSS-induced colitis	Inhibition of elastase and proteinase-3	Bermúdez-Humarán et al., 2015
*L. lactis* NZ9000	Mouse leukocyte protease inhibitor	NICE	Mouse model of DSS-induced colitis	Reduced tissue damage and MPO activity	Bermúdez-Humarán et al., 2015
*L. lactis* NZ9000	Mouse TGF-β	NICE	Mouse model of DSS-induced colitis	Reduced granulocytes infiltration	Bermúdez-Humarán et al., 2015
*L. casei* CECT 5276	Human IL-10 combined with 5-aminosalicylic acid (5-ASA)	Lactose inducible promoter	Mouse model of DSS-induced colitis	Inhibition of NF-κB pathway	Qiu et al., 2013
*L. lactis* MG1363	Mouse IL-10	TREX1	Mouse model of DSS-induced colitis and IL-10 knockout mice	Reduced tissue damage	Steidler et al., 2000
*L. lactis* MG1363	Mouse IL-10	SICE	Mouse model of DNBS-induced colitis	Reduced tissue damage	Benbouziane et al., 2013
*L. lactis* AG013	Human IL-10	ThyA native promoter from *L. lactis*	Clinical trial with Crohn’s disease patients	No significant improvement comparing to placebo	Steidler et al., 2003
*L. lactis* NZ9000	Human pancreatitis-associated protein (Reg3A)	NICE	Mouse model of 5-fluoracil – induced intestinal mucositis	Villous architeture preservation and improved Paneth cells activity	Carvalho et al., 2017
*L. lactis* AG013	Human trefoil factor I	ThyA native promoter from *L. lactis*	Hamsters model of radiation-induced oral mucositis	Reduced clinical scores of oral mucosits	Rottiers et al., 2009
*L. lactis* AG013	Human trefoil factor I	ThyA native promoter from *L. lactis*	Clinical trial with oral mucositis patients	Reduced the severity and course of radiation-induced oral mucositis	Limaye et al., 2013

Lactic acid bacteria have been proven to successfully express proteins of interest in different cell compartments (in the cytoplasm, anchored to the cell membrane or secreted into the extracellular medium) (Miyoshi et al., 2010; Pontes et al., 2011; Pereira et al., 2014). It has been shown that LAB can be administered orally, making the need for clean needles and syringes unnecessary. In fact, the WHO recommends that immunization or treatment be orally administered due to economic, logistical and security reasons. Furthermore, this route offers important advantages over systemic administration, such as reducing side effects, as the molecules are administered locally and have the ability to stimulate the GALT immune responses (Levine and Dougan, 1998; Neutra and Kozlowski, 2006; Bermúdez-Humarán et al., 2011).

The majority of studies in the literature describe the genetic engineering of *L. lactis* because it is the best-characterized member of the LAB group, both physiologically and genetically, and a large number of genetic tools are available for its genetic manipulation. Additional features that make *L. lactis* one of the most extensively studied bacteria are related to its economic importance in cheese production, as it is easy to grow and manipulate and was the first LAB to have its genome completely sequenced (de Vos, 1999; Bolotin et al., 2001; Felis and Dellaglio, 2007; Wells and Mercenier, 2008; Bermúdez-Humarán et al., 2011). In addition, it does not produce endotoxins such as lipopolysaccharide (LPS) and secretes few proteins, facilitating the purification of heterologous proteins. In fact, only the unknown secreted protein of 45 kDa (Usp45) is detectable after sodium dodecyl sulfate polyacrylamide gel electrophoresis (SDS-PAGE) stained with Coomassie brilliant blue (van Asseldonk et al., 1990; Bahey-El-Din et al., 2010).

### *Lactococcus lactis*, the Model Lactic Acid Bacteria for the Expression of Anti-inflammatory Molecules

#### Properties of *L. lactis*

*Lactococcus lactis* is a mesophilic, facultative heterofermentative bacterium with an optimum growth temperature of approximately 30°C that is important in dairy industry, especially for cheese production. There are two reported subspecies (ssp.) of *L. lactis*, ssp. *lactis* and ssp. *cremoris*. Both can be found naturally in plants, especially grass. As they are used in the food industry for milk fermentation, both species can also be found in dairy products, such as cheeses, yogurts, and some breads and wines (Carr et al., 2002). *L. lactis* subsp. *cremoris* MG1363 is the most commonly used strain for cloning and protein expression, as it has no plasmids and does not produce any extracellular proteases. In addition, this strain was cataloged by the FDA and the European Food Safety Authority (EFSA) as a safe microorganism (GRAS), non-invasive and non-pathogenic, reinforcing its use as a factory for the production of anti-inflammatory molecules. Although it is considered GRAS, *L. lactis* spp. *lactis* was reported to cause an infection in two individuals who had been diagnosed with cardiac abnormalities. Afterward, they were treated with antibiotics, and the infection was cleared. Both patients did not develop any further infection by *L. lactis* (Mercenier, 1999; Bermúdez-Humarán et al., 2011). As *L. lactis* does not colonize the human GIT, most studies have focused on the beneficial effects of LAB strains in the *Lactobacillus* genus, which is autochthonous. However, recent studies have demonstrated that some allochthonous lactococci strains have anti-inflammatory properties. Ballal et al. (2015) found that *L. lactis* I-1631 prevents colitis in T-bet-/- Rag2-/- mice. Two additional studies have shown that NCDO2118 sub. *lactis* or FC sub. *cremoris* are anti-inflammatory when inoculated in inflamed mice receiving the chemical agent DSS (Nishitani et al., 2009; Luerce et al., 2014). Moreover, *L. lactis*, was used for the treatment of eosinophilic esophagitis in mice. It was demonstrated that the administration of NCC2287 in mice decreased esophageal eosinophilia, which was elicited by epicutaneous sensitization with protein extract from the fungi *Aspergillus fumigatus*, highlighting the beneficial effects of *L. lactis* in another severe inflammatory disease (Holvoet et al., 2016).

As mentioned previously, there are several expression systems available for heterologous protein production in *L. lactis* (Miyoshi et al., 2010). This has allowed the cloning and expression of different heterologous anti-inflammatory proteins by the use of both cloning and expression vectors designed for *L. lactis* (**Table 1**) (Langella and Le Loir, 1999; Le Loir et al., 2005; Bermúdez-Humarán et al., 2011).

### Heterologous Protein Expression Systems in *L. lactis*

The first expression systems for use in *Lactococcus lactis* were based on the classic bacterial lactose operon. This operon is activated when the lac promoter is induced in the presence of lactose, while the transcriptional repressor gene (*lacR*) is suppressed in the same condition. Therefore, lactococci strains harboring a plasmid carrying this operon fused to a target gene allow recombinant proteins to be expressed in a tightly controlled fashion (van Rooijen et al., 1992). Wells et al. (1993) improved this system by integrating it with a strong phage promoter that allowed for high levels of heterogous protein production. It consisted of three plasmids containing the lac operon elements and two elements from the T7 bacteriophage found in *E. coli*. In this system, the presence of lactose induces the lac promoter in the first plasmid, promoting expression of the T7 RNA polymerase. Afterward, the T7 RNA polymerase activates expression of the gene of interest controlled by the T7 promoter in the second plasmid. The third plasmid coded for the functional lac operon, allowing the cell to be capable of metabolizing soluble lactose in an artificial medium. This system and other complex systems based on phage promoters have allowed for the strict control of gene expression, although they require many antibiotic resistance markers, making them unsuitable for use in the food and pharmaceutical industry (Wells et al., 1993; Nauta et al., 1996; O’Sullivan, 2001).

In this context, several studies have been carried out to develop safer and more simple vectors. One of the most powerful expression systems already developed for use in the food industry is based on genes involved in the biosynthesis and regulation of the antimicrobial nisin, a peptide naturally secreted by several strains of *Lactococcus lactis.* In brief, the Nisin-Controlled Gene Expression system (NICE) is based on the expression of three genes involved in the production and regulation of the the peptide nisin, which is naturally secreted by various *L. lactis* strains, in a genetically engineered *L. lactis* strain. The *nisR* and *nisK genes* encode a two-component regulatory system (NisRK), which controls the expression of the nisin operon through the activation of signal transduction pathways (Kuipers et al., 1993). The strain used in this system is a genetically modified version of a *L. lactis* MG1363 strain, *L. lactis* NZ9000, in which both *nisR* and *nisK* regulatory genes were inserted into its chromosome. The expression vector contains the nisin promoter P*nisA*, followed by multiple cloning sites (MCSs) for the insertion of heterologous genes coding for anti-inflammatory molecules or antigens (Kuipers et al., 1993; Mierau and Kleerebezem, 2005). Because NICE system expression vectors exist in different versions, heterologous proteins can be expressed in different cellular compartments. In addition to the cytoplasm, recombinant protein can be anchored to the bacterial cell wall by means of a cell wall anchor (CWA) peptide, composed of 30 amino acids located in the carboxy-terminal portion (C-terminus) of the protein. CWA is recognized by the cell anchoring machinery and is usually covalently attached to the peptidoglycan from the cell membrane. Furthermore, recombinant proteins may be coupled with a short (5–30 amino acid long) peptide present at the N-terminus region of the heterologous protein, allowing its translocation across the cell membrane and secretion to the extracellular medium (Le Loir et al., 1994; Piard et al., 1997).

The NICE system has been successfully used to express and address a variety of heterologous proteins of medical and biotechnological interest, and according to some authors, it is considered as one of the best genetic tools already developed for gene cloning and expression in *L. lactis* (Nouaille et al., 2003; Le Loir et al., 2005).

Miyoshi et al. (2004) developed the xylose-inducible expression system (XIES) based on the xylose permease gene promoter (PxylT) from *Lactococcus lactis* NCDO2118. In the presence of glucose, fructose and/or mannose, PxylT was shown to be repressed; otherwise, PxylT is transcriptionally activated by xylose in *Lactococcus lactis* (Miyoshi et al., 2004). Therefore, this system could be successively turned on by adding xylose and turned off by washing the cells and growing them on glucose. The system combines the use of PxylT, the ribosome-binding site (RBS) and the signal peptide (SP) of the lactococcal secreted protein Usp45 and the *Staphylococcus aureus* nuclease gene (*nuc*) as the reporter (Shortle, 1983; Le Loir et al., 1994). This system was successfully used for the production of highlevels of Nuc, which was tested for correct protein targeting in the *Lactococcus lactis* subsp. *lactis* strain NCDO2118. These systems are considered less expensive and safer for laboratory use compared to many available expression methods (de Azevedo et al., 2015).

Most heterologous protein expression systems used in *L. lactis* are based on inducible promoters, which allows for the controlled expression of the protein of interest. In this context, they prevent protein aggregation and degradation within the bacterial cytoplasm. However, the majority of the expression vectors present inherent safety drawbacks due to the necessity to add chemical compounds into the bacterial culture to induce heterologous protein expression prior to *in vivo* administration. Other food grade expression systems that do not require the pre-induction of the cultures to allow the expression of a given recombinant protein have been reported (Derre et al., 1999; Ruiz et al., 2012; Benbouziane et al., 2013). Benbouziane et al. (2013) developed the stress-inducible controlled expression system (SICE), based on the use of the heat shock protein groESL operon promoter (pGroESL) from *L. lactis*, to deliver proteins of health interest *in situ*. Heat-shock proteins play an essential role under different stress conditions such as heat-shock, low pH, UV-irradiation, and salt stress. Indeed, upon administration into the host, recombinant bacteria should find very different conditions from culture conditions and likely suffer different types of stress (Benbouziane et al., 2013). In the case of oral administration, heat stress can be accompanied by an acid stress during passage through the stomach as well as bile stress in the duodenum. SICE system represents an interesting alternative for the treatment of GI inflammatory diseases, since it allows for the local delivery of therapeutic proteins in the GIT during the passage of the bacteria, allowing for the localized action of the protein and thus a greater efficiency. This system is an interesting alternative for proof of concept studies because it does not require the presence of regulatory genes or the pre-induction of the cultures. However, it still presents a bottleneck, since antibiotic resistance markers could be horizontally transferred to harmful microbes in the human GIT in clinical studies. In this context, the scientific community has been trying to develop biological confinement strategies, which are discussed later in this review (Vandermeulen et al., 2011).

### Therapeutic Interventions Using Recombinant *L. lactis* Strains to Alleviate GI Inflammation

Since *L. lactis* can be genetically modified to efficiently produce and secrete different anti-inflammatory proteins, recombinant strains of *L. lactis* have been tested in pre-clinical and clinical experimental trials to treat or prevent various human diseases, including intestinal inflammation (**Table 1**) (Steidler et al., 2000; Rochat et al., 2007; LeBlanc et al., 2011; Bermúdez-Humarán et al., 2013; Del Carmen et al., 2014; Carvalho et al., 2016, 2017). The oral administration of *L. lactis* expressing anti-inflammatory proteins is a very interesting strategy to fight GIT inflammation, as this species is non-invasive and allochthonous, as commented on earlier. As it is unable to colonize the GIT, the potential to elicit adverse effects on host microbiota related to its long-term administration is reduced (Nouaille et al., 2003). It has been shown that the oral administration of a recombinant *L. lactis* strain expressing the enzyme SOD, naturally produced by *Bacillus subtilis*, reduced inflammation scores in animals treated with trinitrobenzenesulfonic acid (TNBS). This therapeutic effect was tied to the antioxidant properties of the recombinant SOD (Rochat et al., 2005). Later, the same strain was able to prevent the development of colorectal cancer cells in mice.

In another proof-of-concept study, the anti-inflammatory strain *L. lactis* NCDO 2118 was engineered to produce the oxidative enzyme, 15-lipoxygenase-1 (15-LOX-1), which catalyzes the formation of several anti-inflammatory mediators, such as lipoxins, resolvins and protectins. The 15-LOX-1 produced by *L. lactis* was effective in treating DSS-induced colitis in mice during the remission period and decreased pro-inflammatory cytokines such as IFN-γ and IL-4 while increasing the anti-inflammatory IL-10 (Carvalho et al., 2016). Another strategy has been the use of *L. lactis* to secrete either regulatory cytokines involved in the regulation of inflammation processes, or antibodies that neutralize pro-inflammatory cytokines. *L. lactis* strains able to secrete anti-TNFα antibodies that bind to TNF-α, one of the most important mediators of inflammation, were described (Yoshida and Miyazaki, 2008; Strukelj et al., 2014). It was demonstrated in a DSS-induced colitis mouse model that the oral administration of *L. lactis* expressing murine anti-TNFα showed reduced inflammation, and work by Bermúdez-Humaran and collaborators demonstrated that a recombinant *L. lactis* strain expressing the cytokine TGF-β was able to ameliorate clinical symptoms, such as weight loss and diarrhea in the same DSS model of intestinal inflammation (Yoshida and Miyazaki, 2008; Bermúdez-Humarán et al., 2015). Another strain that is presenting good results in pre-clinical trials expresses IL-10, an anti-inflammatory cytokine capable of suppressing proinflammatory responses of both innate and adaptive immune cells. The effect of the recombinant IL-10 producing *L. lactis* has been tested in several IBD animal models, such as IL-10 knockout mice and TNBS or DSS models (Steidler and Schotte, 2000; Steidler et al., 2003; Braat et al., 2006; Del Carmen et al., 2014). The recombinant IL-10 producing *L. lactis* strain demonstrated promising results in pre-clinical. Indeed, a large clinical trial using recombinant *L. lactis* secreting the human IL-10 was conducted in patients with Crohn’s disease approximately 10 years ago. Its use in humans was allowed by regulatory agencies, such as the Genetically Modified Organisms (GMOs) European Commission, because of a biological containment strategy that was developed. A gene encoding the essential protein thymidylate synthase (ThyA), located on the *L. lactis* chromosome, was exchanged for the human IL-10 gene. Therefore, the strain was only able to survive in the presence of thymine or thymidine that was artificially provided in the culture medium, making *L. lactis*-IL-10 critically dependent on this compound. Inside the human body, the strain could survive and deliver IL-10, since thymine or thymidine is available. Outside of the body, the GMO strain was unable to survive, avoiding its spread into the environment (Steidler et al., 2003). Clinical results showed no significant improvement between patients receiving the IL-10 producing *L. lactis* strain and those who received a placebo (Braat et al., 2006).

Few studies regarding the treatment of mucositis using recombinant *L. lactis* strains expressing therapeutic molecules have been reported. Most pre-clinical studies found in the literature describe the use of purified anti-inflammatory compounds intended to eliminate disease. An example is the systemic administration of either IL-11 or TGF-β regulatory cytokines in patients. The authors noted that this alternative treatment was not able to contain oral mucositis. The possible causes for this failure were linked to an inadequate dosage, route of administration and drug stability (Antin et al., 2002; de Koning et al., 2006). Other clinical studies have tested growth factors that stimulate cell proliferation, thereby maintaining epithelial barrier integrity, such as granulocyte-macrophage colony-stimulating factor (GM-CSF) and epidermal growth factor (EGF). However, their use was associated with an increased risk and progression of tumors (Hong et al., 2009). Rottiers et al. (2009) evaluated the effect of *L. lactis* secreting trefoil factor I (TFF-1), naturally involved in the repair of the epithelial barrier, administered to hamsters with oral mucositis. It was observed that recombinant *L. lactis* was able to reduce mucosal inflammation (Rottiers et al., 2009; Caluwaerts et al., 2010). Furthermore, as undesired reactions were not detected in pre-clinical trials, another genetically modified *L. lactis* strain (AG013), capable of secreting human TFF1, was engineered based on the ThyA biological confinement system. A phase 1 clinical trial was performed in patients with oral mucositis who tolerated the treatment well, and administration of the AG013 strain was shown to be more efficient in ameliorating clinical syntoms than placebo (Limaye et al., 2013). Several molecules with anti-inflammatory properties have sought to be cloned and expressed in *L. lactis*, which has proven to be a safe vehicle for the treatment of GI intestinal disorders. Anti-inflammatory cytokines, anti-oxidant enzymes, epithelial growth factor and especially antimicrobial peptides produced by *L. lactis* are the focus of future research efforts for the development of a possible treatment for GI tract inflammation.

#### Mammalian Antimicrobial Peptides Produced by *L. lactis* as a Possible Treatment for Intestinal Inflammation

Antimicrobial peptides that are involved in the maintenance of the epithelial barrier could represent an interesting candidate to prevent microbiota-driven inflammatory signaling. Various antimicrobial peptides, such as defensins, cathelicidins and histatins, that are produced by Paneth cells seem to play a critical role in intestinal homeostasis, and their biological activity has been reported to be compromised in IBD patients (Clevers and Bevins, 2013; Peterson and Artis, 2014). Different research groups are investigating whether the administration of these peptides could have a protective effect against intestinal inflammation. In a study conducted by Seo et al. (2012), α-defensin (HD5) and human β-defensin 2 (HBD2), which have been purified from the probiotic *E. coli* Nissle 1917, inhibithed the growth of pathogenic *E. coli*, *S. typhimurium*, or *L. monocytogenes* when co-incubated, *in vitro*, with these bacterial species (Seo et al., 2012).

Another antimicrobial peptide, cathelicidin, was expressed in *L. lactis* and the efficacy of this strain in decreasing intestinal inflammation was evaluated in a DSS murine model. The authors observed a reduced number of bacteria in the feces from animals that received the *L. lactis*-cathelicidin strain, suggesting an anti-microbial effect of the strain. According to the study, these findings were correlated to reduced tissue damage and MPO activity (Wong et al., 2012).

Among the antimicrobial peptides, the C-type lectin, Reg3A has been extensively studied due to its protective effect in the intestines of humans and animals during the inflammation process. This peptide, also known as pancreatitis-associated protein (PAP), belongs to the Reg family, which encodes a diverse group of proteins called secreted C-type lectins that contain a carbohydrate recognition domain (CRD). The Reg3A protein is predominantly produced in the small intestine of mammals, mainly by Paneth cells, where the density of microorganisms is higher (Christa et al., 1996). Several studies revealed that Reg3A exerts a bactericidal activity against Gram-positive bacteria. Furthermore, it appears that its activation in the intestinal mucosa is required to generate a protective response against intestinal microbiota during bacteria-driven inflammatory events (Christa et al., 1999; Malka et al., 2000). In fact, the PAP protective effect in GI inflammation models has been demonstrated for the first time in a DSS-induced colitis rat model. This work used an adenovirus strategy to deliver PAP cDNA into host cells to increase the expression of PAP (Lv et al., 2012). Recently, Breyner et al. (2017, personal communication) have shown that the use of *L. lactis* expressing human PAP could prevent colitis in a DNBS-chemically induced murine model. Interestingly, as it was shown to be useful in the treatment of IBD, another study sought to investigate a protective role of *L. lactis* secreting human PAP in mucositis using the 5-FU-induced intestinal mucositis experimental mouse model. The authors showed that the PAP antimicrobial peptide, cloned into *L. lactis*, has an inhibitory effect against the opportunistic commensal *E. faecalis*. Moreover, *L. lactis* NZ9000 by itself was able to prevent histological damage and reduce neutrophil and eosinophil infiltration in mice injected with 5-FU. In addition, the recombinant lactococci producing PAP improved villous architecture preservation and increased Paneth cell activity in response to 5-FU inflammation (Carvalho et al., 2017).

## Conclusion

The efficacy of probiotic LAB, especially in the context of using recombinant *L. lactis* strains designed to deliver anti-inflammatory proteins *in situ*, has been demonstrated for treating IBD in many studies in the past decades. Moreover, as highlighted in this review, the same therapeutic approach is being successfully transposed for treating mucositis. Thus, this work reiterates that probiotic LAB, wild type or genetically modified, could also be used as an alternative for treating other GI inflammatory diseases in which dysbiosis has been shown to be implicated. As most of the beneficial effects of recombinant *L. lactis* strains have been demonstrated in proof-of-concept studies, further translational aproaches are needed to make them safe for testing in humans. In this context, biological confinement strategies that prevent recombinant lactococci from escaping into natural ecosystems should be considered.

## Author Contributions

RC contributed to conception of the work, bibliographic survey, and manuscript writing. FdC contributed to bibliographic survey and drafting or the work. AdO was responsible for creating the figure and contributed to the bibliographic survey. PL was involved in the critical revision of the article. J-MC was involved in the critical revision of the article. LB-H was involved in manuscript correction and drafting of the work. VA contributed to critical revision of the article and conception of the work. MdA contributed to manuscript writing and correction.

## Conflict of Interest Statement

The authors declare that the research was conducted in the absence of any commercial or financial relationships that could be construed as a potential conflict of interest.
